# The role of bovine pericardium matrix in immediate prepectoral breast reconstruction: Insights from the largest published retrospective series

**DOI:** 10.1016/j.jpra.2025.09.005

**Published:** 2025-09-11

**Authors:** M. Nogueira Sixto, G. De Castro Parga, M.J. Lamas González, M.R. Melendez Villar, V. Rodríguez Fernandez, Z. Valladares Bajo, G. Freiria Barreiro, R. Mallo Alonso

**Affiliations:** Breast Pathology Unit, Complexo Hospitalario Universitario de Vigo (CHUVI), Hospital Meixoeiro, Estrada de Madrid 145, 36214 Vigo, Pontevedra, Galicia, Spain

**Keywords:** Inmediate prepectoral breast reconstruction, Prepectoral reconstruction, Bovine pericardium matrix, Biologic mesh, Outcomes

## Abstract

Immediate prepectoral breast reconstruction (IPBR) with biological matrices is a widely adopted technique, traditionally guided by ABS-BAPRAS eligibility criteria. In Europe, their use has declined in recent years, while polyurethane-covered implants have emerged as an alternative in some centers. This study reports the outcomes of IPBR with Exaflex, a biological matrix, performed at CHUVI in a real-world clinical setting.

A retrospective analysis was conducted on a cohort of 80 patients and 112 mastectomies from a prospectively maintained database. The mean patient age was 51 years, and the average BMI was 24.3 kg/m2; 21.25 % had a BMI <21 and 18.75 % were smokers. Preoperative MRI showed a pinch test >15 mm in 68.75 % of breasts. Preoperative radiotherapy was administered in 2.67 % of breasts, and 32.1 % received postoperative radiotherapy. 43.75 % of patients presented with two or more concurrent risk factors Nipple-sparing mastectomy (NSM) was the most common technique (92.9 %), and all reconstructions were prepectoral. Mean breast weight was 212.8 g and implant volume 327 cc. Axillary surgery was performed in 59 % of cases (SLNB 55.4 %, ALND 5.3 %).

Complication rates included seroma (5.4 %), hematoma (8 %), wound infection (6.25 %), prosthetic infection (4.46 %), skin necrosis (7.14 %), rippling (9.8 %), and capsular contracture (2.67 %). No animation deformities were observed. Aesthetic reoperation occurred in 10.7 % of cases, and reconstruction failure in 1.78 %. Mean follow-up was 13.5 months (median 13). A statistically significant association was observed between postoperative radiotherapy and capsular contracture (*p* = 0.03), while no significant association was found between radiotherapy and prosthetic infection (*p* = 0.32), skin necrosis (*p* = 0,71) or rippling (*p* = 0.47). No significant association was found between any individual risk factor (diabetes, low/high BMI, smoking, radiotherapy, or pinch thickness <15 mm) and the occurrence of complications. Although the number of risk factors per patient (0, 1, 2, or ≥3) was not significantly associated with complication rates, a non-significant trend toward increased risk was noted among patients presenting multiple concurrent factors.

**Conclusion:**

IPBR with Exaflex is a safe and reproducible technique with favorable outcomes. The ABS-BAPRAS criteria are highly selective. However, despite the limitations of our study, our findings suggest that, in experienced hands, these criteria might be cautiously expanded to include NSM patients with selected risk factors.

## Introduction

**Figure 1:**
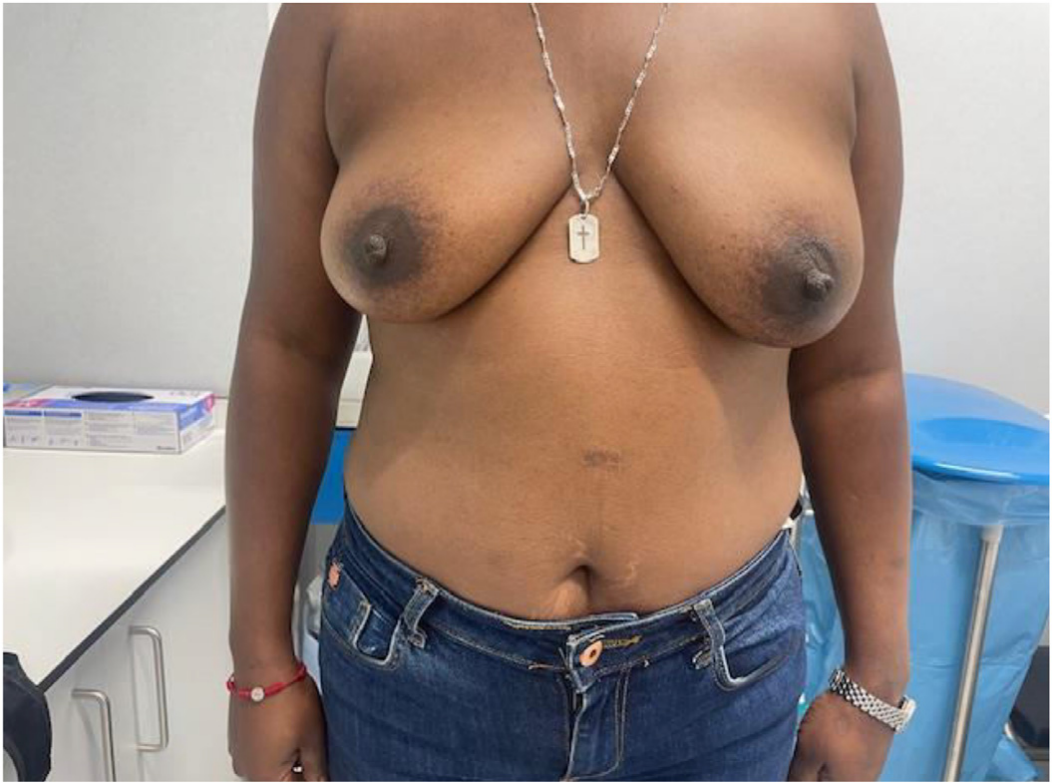
Preoperative frontal view of Case 1.

**Figure 2:**
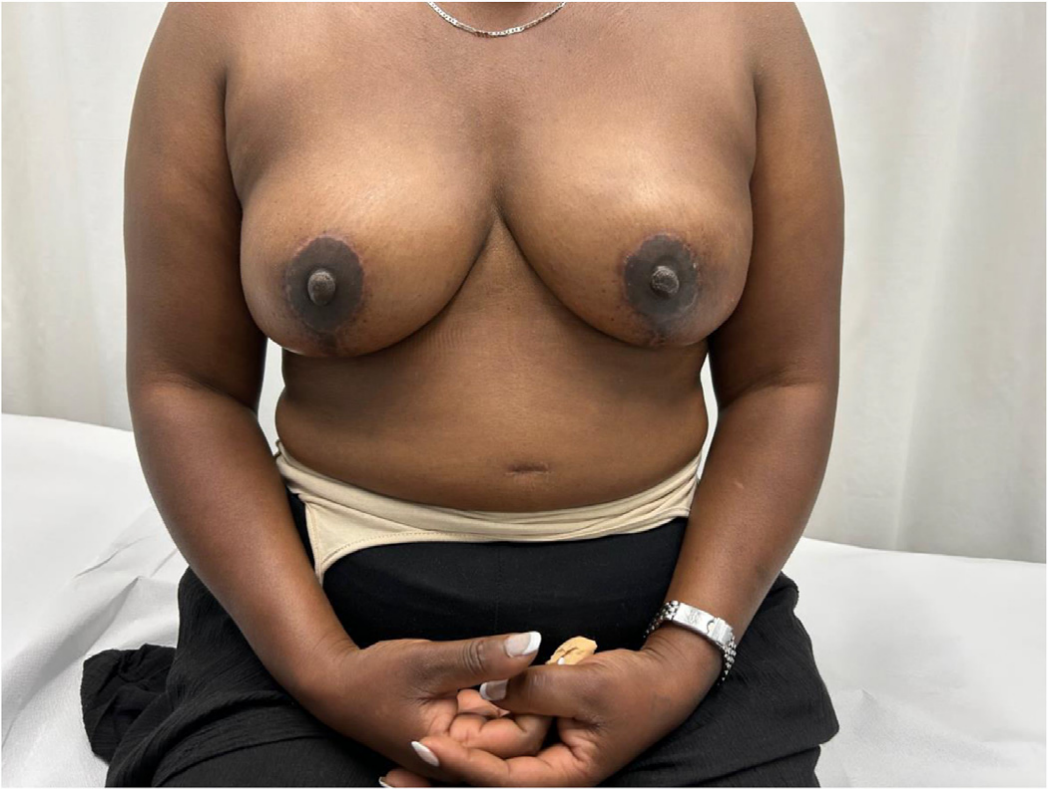
Postoperative frontal view of Case 1.

**Figure 3:**
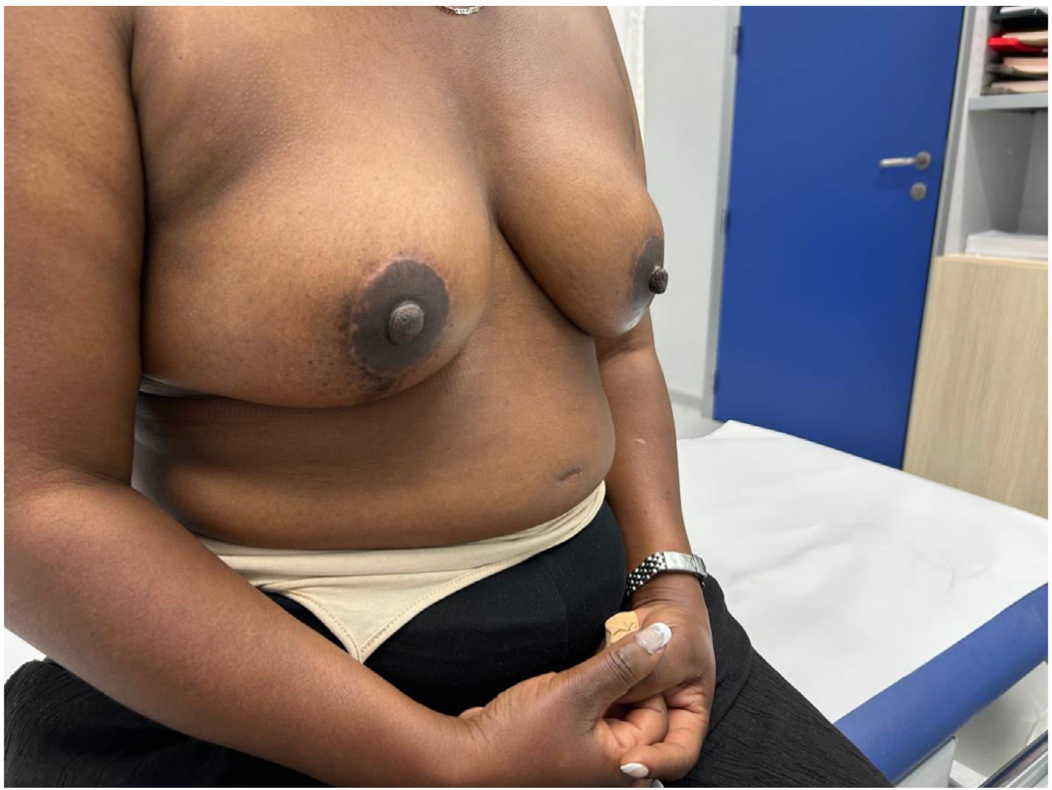
Postoperative lateral view of Case 1.

**Figure 4:**
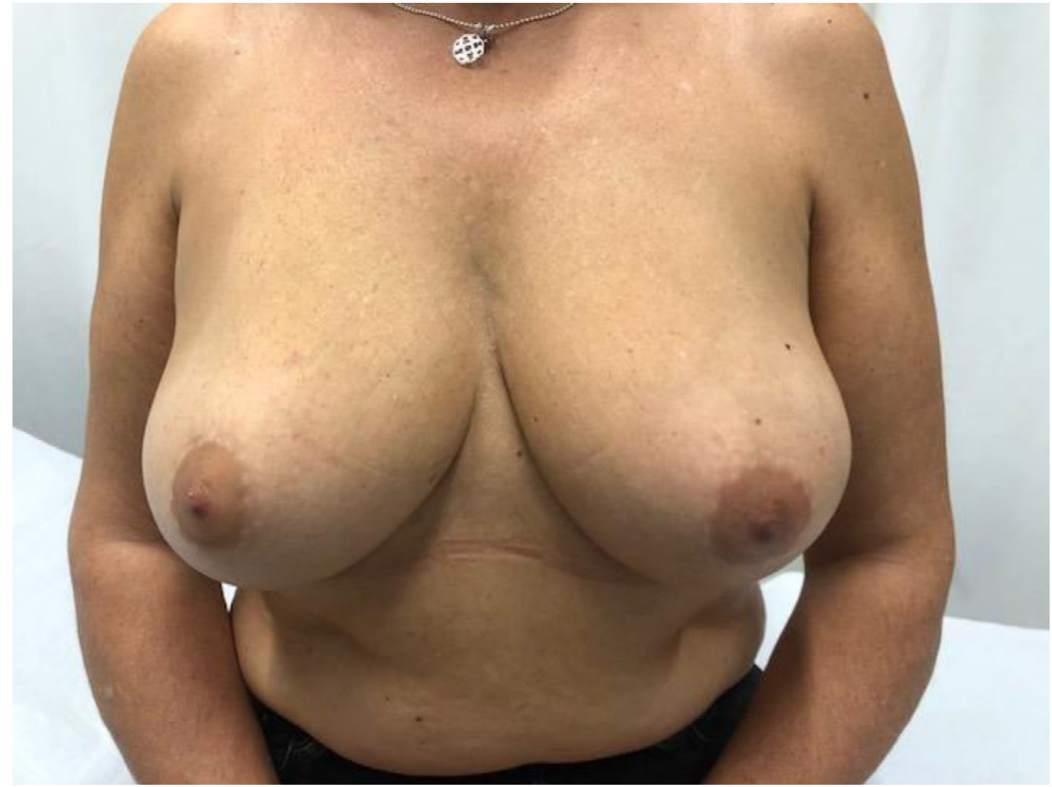
Preoperative frontal view of Case 2.

**Figure 5:**
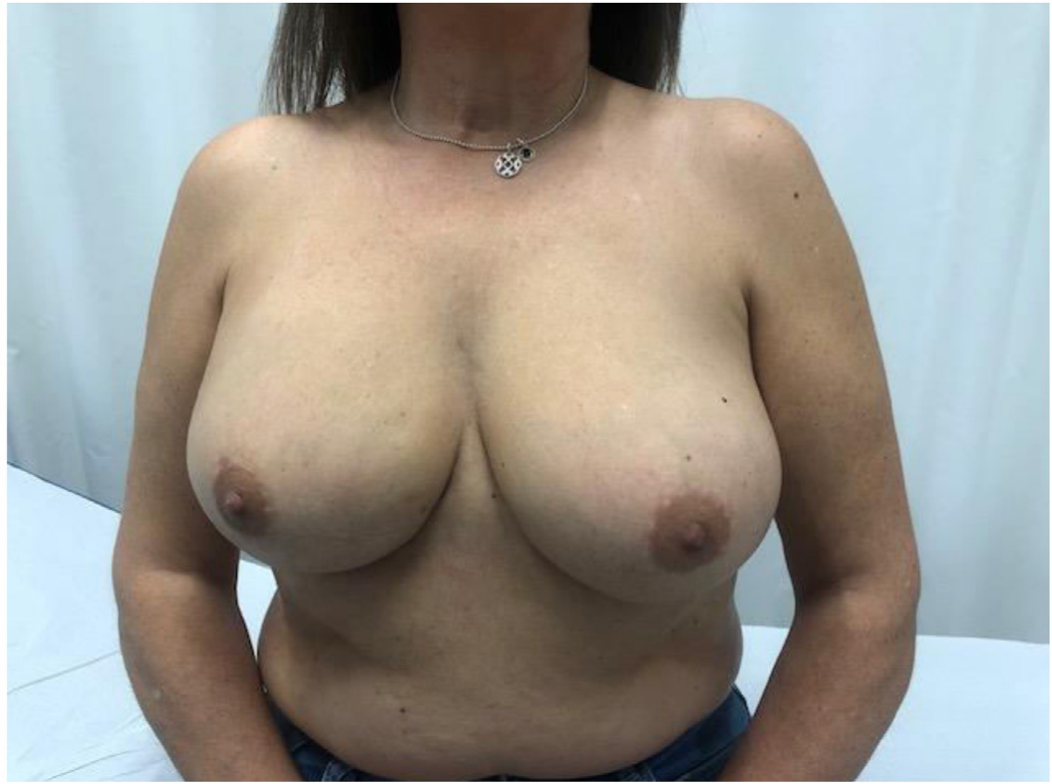
Postoperative frontal view of Case 2.

Advances in skin flap vascularization, surgical techniques and intraoperative technologies over the past few decades have made it possible to recover the prepectoral plane during immediate post-mastectomy reconstruction. A key milestone in the adoption of the prepectoral plane was the introduction of acellular dermal matrices (ADMs). Their theoretical benefits include creating a biologically favorable environment around the implant that reduces local inflammation and, consequently, the risk of capsular contracture. ADMs also improve skin support by limiting tissue stretching, which helps prevent bottoming-out and recurrent ptosis, while adding an additional layer between the implant and skin flap to reduce rippling^1^ (Figure 1, Figure 2, Figure 3, Figure 4–5).

Multiple series, clinical trials, and reviews support the favorable outcomes of prepectoral ADM use in both oncologic and prophylactic mastectomies. Although various ADMs and biological matrices are available, they are commonly classified by origin: human (hADM), porcine (pADM), or bovine (bADM).^2^ To date, none have demonstrated superior outcomes compared to their counterparts. In the USA, hADMs are the most widely used; in Europe, pADMs—particularly Braxon®—are the most commonly adopted. The use of hADMs in Europe remains limited, mainly due to current regulatory restrictions.^2^

In this context, we present our single-centre experience using the Exaflex® bovine pericardial mesh on patients who do not strictly meet the ABS/BAPRAS criteria, but who are representative of those seen in routine clinical practice in our setting (Spain, Europe). Additionally, we aim to demonstrate that the favorable outcomes reported in other series can be reproduced with biological meshes of different origin and manufacturer. Alongside these developments, polyurethane-covered implants have more recently been investigated as an alternative approach in prepectoral reconstruction. While some institutional series have reported encouraging results, their use remains limited to selected centers, and no population-level data are yet available to confirm widespread adoption.

## Material and methods

A prospectively maintained database was conducted for a retrospective cohort of all patients who have undergone oncological or prophylactic mastectomy with immediate prepectoral reconstruction with Exaflex bovine pericardial biological matrix. Nipple-sparing, skin-sparing and skin-reducing mastectomies were included. All mastectomies and subsequent immediate reconstructions - regardless of whether postoperative radiotherapy was planned - were performed at a single institution by the same team of seven board-certified breast surgeons belonging to the Breast Pathology Unit. Data were obtained from a prospectively maintained institutional database, which helped minimize selection bias inherent to retrospective analyses.

Patient selection followed the ideal criteria proposed by ABS/BAPRAS^3^, however, BMI >30, diabetes, smoking, and perioperative radiotherapy (RT) were not considered absolute contraindications unless combined with additional risk factors. Flap quality was assessed preoperatively using two methods: a pinch test during consultation and MRI evaluation based on the criteria described by Radu et al.^4^. Intraoperatively, flap quality was assessed by evaluating vascular perfusion, bleeding, and the absence of venous congestion.

Patients were closely monitored, and data were collected on their demographics, medical history, and surgical details (Table 1), as well as postoperative complications, including: hematoma, seroma, wound dehiscence, skin necrosis, wound infection, prosthetic infection, implant extrusion, capsular contracture, rippling, animation deformity, need for reoperation, and implant loss (Table 2). Definitions of each complication are provided in Annex 1.

**Table 1:** Population descriptive.

Characteristic	Value
Total number of patients	80
Total number of breast	112
Mean follow-up (months)	13.5
Median follow-up (months)	13
Mean age (years)	51
Mean BMI (kg/m²)	24.31
BMI (kg/m²) > 30	8 (10 %)
BMI (kg/m²) < 21	17 (21.25 %)
Smokers (patients)	15 (18.75 %)
Pinch > 10 mm pre-surgical MRI (breasts)	112 (100 %)
Pinch 10–15 mm pre-surgical MRI (breasts)	35 (31.25 %)
Prophylactic mastectomies (breasts)	38 (33.9 %)
Preoperative radiotherapy (patients)	2 (2.5 %)
Preoperative radiotherapy (breasts)	3 (2.67 %)
Postoperative RT (patients)	33 (41.25 %)
Postoperative RT (breasts)	36 (32.14 %)
Most frequent mastectomy type (breasts)	Nipple-sparing: 105 (92.8 %)
Type of reconstruction (breasts)	Prepectoral: 112 (100 %)
Mean surgical time (min)	128
Mean breast weight (g)	212.96
Mean prosthetic volume (cc)	328
Breasts with axillary surgery	64 (57.14 %)
– Sentinel lymph node biopsy (SLNB)	58 (51.78 %)
– Axillary lymphadenectomy	6 (5.35 %)
Risk factors (patients)	
No risk factors	21 (26.25 %)
1 risk factor	24 (30 %)
2 risks factors	17 (21.25 %)
≥ 3 risks factors	18 (22.5 %)

**Table 2:** Outcomes.

Outcome	Frequency (%)
Seroma	6 (5.35 %)
Hematoma	9 (8.03 %)
Wound infection	7 (6.25 %)
Prosthetic infection	5 (4.46 %)
Skin necrosis	8 (7.14 %)
Rippling	9 (8.03 %)
Capsular contracture	3 (2.67 %)
Animated deformity	0 (0 %)
Reconstruction failure	2 (1.78 %)
Reoperation for aesthetic purposes	12 (10.71 %)

A post-hoc power analysis was conducted to evaluate whether the sample size was sufficient to detect clinically relevant complication rates, defined as proportions ≥10 % and ≥5 %. This analysis was based on a one-sample z-test for proportions, with power calculated according to the observed complication rates. Additionally, 95 % confidence intervals (CIs) for each complication were computed using the Wilson score method to assess statistical precision (Table 3).

**Table 3:** Complication rates and post - hoc analyses.ods.

Complication	Observed Rate ( %)	95 % CI	Power to Detect ≥10 %	Power to Detect ≥5 %
**Prosthetic infection**	4.46	1.92 – 10.03	63.5	5.8
**Capsular contracture**	2.67	0.92 – 7.58	91.6	25.5
**Rippling**	8.03	4.29 – 14.57	11.3	25.8
**Haematoma**	8.03	4.29 – 14.57	11.3	25.8
**Seroma**	5.4	2.48 – 11.2	45.5	5.4
**Wound infection**	6.25	3.06 – 12.34	31.0	8.9
**Reconstruction failure**	1.78	0.49 – 6.28	97.8	49.2
**Animated deformity**	0.0	0.0 – 3.32	100.0	99.8

To explore associations between postoperative complications and factors such as prophylactic mastectomy, postoperative radiotherapy, or preoperative pinch thickness of <15 mm, Fisher’s exact test and odds ratios were used (Table 4). We also examined whether individual risk factors (diabetes, BMI <21 or >30, smoking, radiotherapy, and pinch <15 mm) (Table 5) or their cumulative number (0, 1, 2, or ≥3) (Table 6) were associated with complications. Chi-square tests were applied for categorical comparisons, and Fisher’s exact test used when expected cell counts were low.

**Table 4:** Comparison of complication rates in subgroups.

Complication	Cases in Prophylactic	Cases in Non-Prophylactic	Odds Ratio (CI 95 %)	p-value (Fisher)	10–15 mm cases	>15 mm cases	Odds ratio (CI 95 %)	p-value (Fisher).1	Cases in Postop RT	Cases in No Postop RT	Odds Ratio (CI 95 %)	p-value (Fisher)
Seroma	2	4	0,97 (0.17 - 5.56)	1	0	6	0 (0.01 - 3.11)	0.1743	–	–	–	–
Hematoma	1	8	0,22 (0.03 - 1.85)	0.1638	4	5	1,86 (0.47 - 7.39)	0.4572	–	–	–	–
Wound infection	2	5	0,77 (0.14 - 4.15)	1	0	7	0 (0.01 - 2.59)	0.0962	–	–	–	–
Prosthetic infection	0	5	0 (0.01 - 3.41)	0.1645	0	5	0 (0.01 - 3.87)	0.3225	3	2	3,36 (0.54 - 21.09)	0.3256
Skin necrosis	2	6	0,63 (0.12 - 3.28)	0.7142	3	5	1,35 (0.3 - 5.99)	0.7033	3	5	1,29 (0.29 - 5.73)	0.7103
Rippling	6	3	4,44 (1.04 - 18.87)	0.0596	2	7	0,61 (0.12 - 3.08)	0.7176	4	5	1,78 (0.45 - 7.05)	0.4652
Capsular contracture	1	2	0,97 (0.09 - 11.08)	1	1	2	1,1 (0.1 - 12.58)	1	3	0	inf (0.67 - 283.62)	0.0313
Animation deformity	0	0	–	1	0	0	(0.04 - 113.15)	1	–	–	–	–
Reconstruction failure	0	2	0 (0.02 - 10.77)	0.5476	0	2	0 (0.02 - 12.19)	1	2	0	inf (0.39 – 203.55)	0.1014

**Table 5:** Risk Factors and Postoperative Complications.

Risk Factor	Patients with Risk Factor	Complications in Risk Group	p-value	Test Used
Diabetes	3	1	0.607	Fisher
BMI <21	17	3	0.773	Chi-square
BMI >30	8	2	0.641	Fisher
Smoking	15	2	0.999	Chi-square
Radiotherapy	33	7	0.841	Chi-square
Pinch <15mm	24	4	0.927	Chi-square

**Table 6:** Cumulative Number of Risk Factors.

Number of Risk Factors	Number of Patients	Patients with Complications	Chi-square
0	26	8	
1	28	8	
2	15	5	
≥3	11	6	
p-value			0.956

All statistical analyses were performed using the latest version of IBM SPSS Statistics (IBM Corp., Armonk, NY, USA).

This study was conducted and reported in accordance with the STROBE guidelines for cohort studies.

### Surgical technique

At our center, we perform immediate prepectoral reconstructions using Exaflex® biological mesh following skin- or nipple-sparing mastectomies on patients with small to moderate-sized breasts, especially those at risk of reconstructive failure or with poor pinch test results. This procedure is only performed if the skin flaps are well vascularized. For patients with moderate to large breasts and grade II or III ptosis undergoing skin-reducing or extended skin-sparing mastectomies, we typically use a dermal-fat flap to partially cover the implant. Biological meshes are only used in cases where the patient presents with two or more risk factors (e.g. smoking and a BMI greater than 30).

The Exaflex® avoids the use of chemicals and uses a freeze-drying process, which, in theory, facilitates the integration and formation of the periprothesic capsule. Following a minimum of five minutes of hydration of the matrix , the prosthesis is to be covered with the biological matrix and inserted into the prepectoral pocket. The biological matrix was left unfixed within the pocket. All reconstructions utilized MESMO® implants (Polytech®, Sublime Line), with shape and projection tailored individually. It is standard practice to utilize a siliconized drain under vacuum. All axillary lymph node dissections were performed in nipple-sparing mastectomies with an inframammary incision, requiring a second separate incision for axillary access.

## Results

A total of 80 patients and 112 mastectomies were included, with a mean follow-up duration of 13.5 months and a median of 13 months. The mean patient age was 51 years, and the mean BMI was 24.31 kg/m², with 8 patients (10 %) having a BMI >30, 17 patients (21.25 %) having a BMI <21 and 15 patients (18.75 %) being active smokers. All 80 patients (100 %) had a preoperative MRI-measured pinch thickness >10 mm, although 35 breasts (31,25 %) had a pinch thickness between 10 and 15 mm. 38 mastectomies (33.9 %) were prophylactic, and 3 breasts (2.67 %) had received preoperative radiotherapy. Postoperative radiotherapy was administered to 33 patients (41.25 %), involving 36 reconstructed breasts (32.14 %). All patients received a standard regimen of 40.05 Gy in 15 fractions of 2.67 Gy, except for one case in which a concomitant integrated boost was delivered, reaching 48 Gy in 15 fractions of 3.2 Gy. 45 patients had one or no identifiable risk factors (56.25 %), while 35 (43.75 %) presented with two or more concurrent risk factors (Table 1).

Nipple-sparing mastectomy was the most common procedure, performed in 105 breasts (92.8 %). All reconstructions were immediate, prepectoral, and performed using a direct-to-implant approach. The mean surgical time was 128 min. The mean breast weight was 212.96 g, and the mean implant volume was 328 cc. Axillary surgery was performed in 64 breasts (57.14 %), including sentinel lymph node biopsy in 58 breasts (51.78 %) and axillary lymph node dissection in six breasts (5.35 %) (Table 1).

Postoperative complications included seroma in six reconstructed breasts (5.35 %), hematoma in 9 (8.03 %), wound infection in 7 (6.25 %), prosthetic infection in 5 (4.46 %), skin flap necrosis in 8 (7.14 %), rippling in 9 (8.03 %), and capsular contracture in three cases (2.67 %). No cases of animation deformity were observed. Prosthetic infection was identified in 5 breasts (4.46 %), all of which required intravenous antibiotic therapy. Three cases were managed with implant exchange, while in the remaining two cases, reconstruction ultimately failed, accounting for all cases of reconstructive loss (1.78 %). A total of 12 breasts (10.71 %) underwent further surgical procedures for aesthetic enhancement, including 9 lipofilling procedures to address rippling and three implant exchanges with capsulotomy to address capsular contracture. All revision procedures successfully achieved the intended aesthetic improvement (Table 2). A post-hoc power analysis was performed. The study showed high power (≥90 %) to detect a 10 % rate of capsular contracture (91.6 %), reconstruction failure (97.8 %), and animated deformity (100 %). For animated deformity, the power remained excellent even at the 5 % threshold (99.8 %), supporting the robustness of the zero-incidence observed. However, the remaining complications did not achieve sufficient statistical power to reliably detect event rates at the 10 % or 5 % thresholds. In addition to the post-hoc power analysis, 95 % confidence intervals (CIs) were calculated for the observed complication rates using the Wilson score method (Table 3). A subgroup analysis was performed comparing prophylactic and non-prophylactic mastectomies. Although lower rates of certain complications such as prosthetic infection and hematoma were observed in the prophylactic group, no statistically significant differences were found. A subgroup analysis based on preoperative MRI pinch thickness showed no statistically significant differences in complication rates between breasts with 10–15 mm and ≥ 15 mm thickness. The association between postoperative radiotherapy and capsular contracture reached statistical significance (*p* = 0.03) but no significant associations were found with prosthetic infection (*p* = 0.32), rippling (*p* = 0.47), or skin necrosis (*p* = 0.71) (Table 4). When comparing the presence or absence of complications across patients with 0, 1, 2, or ≥3 risk factors, no statistically significant association was found (*p* = 0.143) (Table 6). Similarly, none of the individual risk factors showed a significant association with overall complications when analyzed separately using Chi-square or Fisher’s exact tests (Table 5).

## Discussion

Current evidence suggests that the prepectoral plane offers superior cosmetic outcomes, reduced postoperative pain, and less functional impairment compared to retromuscular or dual-plane techniques.^5^, ^6^, ^7^, ^8^, ^9^ Multiple studies, including the review by Tellarini et al.^10^ have reported favorable results with the use of biological matrices in prepectoral breast reconstruction. Early comparisons between reconstructions with and without ADMs showed that the ADM group had lower rates of overall complications and capsular contracture.^11^, ^12^, ^13^, ^14^ These findings contributed to the widespread adoption of ADMs and spurred significant industry efforts to develop increasingly advanced products. ADMs are commonly classified by origin: porcine (pADM), bovine (bADM), or human (hADM). To date, no specific subtype or brand has demonstrated clear superiority over the others.^2^ According to the ABS/BAPRAS criteria, patients who are considered suitable candidates for ADM-based reconstruction are those who meet the following characteristics: absence of significant comorbidities (particularly vascular disease or diabetes), small to medium breast size, grade I–II ptosis, normal BMI, a pinch test greater than 1 cm, and an intact pectoralis major muscle. Additionally, smoking, postoperative radiotherapy, and an estimated mastectomy weight over 600 g are listed as risk factors that may negatively impact outcomes.^3^ Although the ideal candidate would meet all the selection criteria, it is common in routine clinical practice to encounter patients who fail to meet at least one of them. Our series included patients with DM, pinch <15 mm, BMI >30 or <21, active smokers, and those receiving perioperative RT (Table 1). These inclusion criteria reflect real-world conditions and supporting the reproducibility of our results in other centers. Due to the high cost of ADMs, some authors have evaluated the results for patients who underwent immediate prepectoral reconstruction without the use of biological matrices.^1^^,^^15^ These studies involve highly selected patients in whom prosthetic coverage is achieved using dermal-fat flaps and/or the placement of tissue expanders, typically in the setting of skin-sparing or skin-reducing mastectomy.^16^, ^17^, ^18^ The dermal-fat flap is an autologous tissue that provides additional protection to the reconstruction, enhancing local vascularization and reducing the risk of implant exposure in the event of wound dehiscence or partial skin flap necrosis. The findings of Dave et al. further support the protective role of the dermal-fat flap: although a higher volume of glandular resection is generally associated with increased complication rates, their study identified mastectomies > 1000 g as a protective factor. This apparent paradox can be explained by the possibility of preserving substantial dermal-fat flaps in these cases, which provide better coverage of the implant. The authors of this publication agree that, in such cases, the use of biological matrices is unnecessary. However, we consider that patients undergoing mastectomy in whom a dermal-fat flap cannot be harvested to cover the implant may benefit from biological matrices, especially when one or more of the previously mentioned risk factors are present.

Several studies have reported the use of ADMs in two-stage prepectoral reconstruction with expanders following skin-sparing, skin-reducing, or nipple-sparing mastectomy.^21^, ^22^, ^23^ In this context, Nolan et al. conducted a critical review of the role of ADMs and questioned their utility in prepectoral reconstruction.^24^ Their analysis compared outcomes of prepectoral expander-based reconstruction with and without ADMs, reporting similar results in both cohorts. Preclinical studies suggest that biological matrices may reduce skin compliance and elasticity, potentially limiting their suitability when combined with tissue expanders. In contrast, the expander alone is often sufficient to maintain the reconstruction and prevent complications. When intraoperative findings raise significant doubts about the feasibility of reconstruction—even with expander placement—conversion to a retromuscular approach or delayed reconstruction may be more appropriate. Based on this evidence, we consider biological matrices particularly useful in immediate prepectoral reconstruction with a definitive implant, rather than tissue expanders.

Although several large series have evaluated the use of different ADMs in prepectoral reconstruction - notably the iBAG study by Masià et al.^19^—to our knowledge, this is the largest published series focused specifically on the use of bovine pericardial matrix.^20^ The outcomes observed in our cohort are in line with those reported in studies using other biological matrices.^10^ As described in previous publications, rippling remains the primary limitation of this technique, although it can typically be corrected with one or more sessions of autologous fat grafting. Notably, our study encompassed a broader patient profile, including 15 active smokers (18.75 %), eight patients (10 %) with a BMI >30, 15 (21.25 %) with a BMI <21, and 36 reconstructed breasts (32 %) exposed to postoperative radiotherapy. When stratified by the number of risk factors, 35 patients (43.75 %) presented with two or more concurrent risk factors, whereas only 21 (26.25 %) had none. Despite these risk factors, our results remained comparable to those of previously published series. In this context, the higher mean implant volume compared with mastectomy weight is probably related to the prevalence of bilateral procedures in our series, together with the routine surgical tendency to upsize implants for improved symmetry and aesthetics. The post-hoc analysis confirmed that the study was adequately powered to detect clinically meaningful rates (≥10 %) of capsular contracture, reconstruction failure, and animation deformity. However, there was insufficient statistical power for the remaining complications, particularly at the lower thresholds (≥5 %). Therefore, while the low rates observed in some complications are supported by statistical robustness, others should be interpreted with caution. While most cases of capsular contracture have been reported to occur within the first year, as shown by Kokosis et al. and Young et al., delayed presentations are not uncommon. Consequently, a longer follow-up is likely to reveal a higher incidence in our cohort. Although prophylactic mastectomy was not significantly associated with fewer complications, the lower event rates suggest a possible protective effect. No significant differences were found between breasts with 10–15 mm and ≥ 15 mm pinch thickness, supporting the 10 mm threshold proposed by Radu et al. Whereas most comparisons did not yield statistically significant associations between individual or combined risk factors and complications, a clinically observed - but statistically non-significant - trend toward higher complication rates was noted in patients with multiple concurrent risk factors (Table 6). Importantly, statistical significance was only reached for the association between postoperative radiotherapy and capsular contracture (*p* = 0.03). Such association are consistent with previous evidence highlighting the adverse effect of radiotherapy in breast reconstruction. No statistically significant associations were found for risk factors such as diabetes, smoking, BMI <21 or >30, pinch < 15 mm, or radiotherapy in relation to other complications (Table 4, Table 5). The overall complication rates in our cohort were low and clinically manageable, reinforcing the safety of this approach. These results suggest that, in experienced hands, IPBR with Exaflex may be safely extended to selected patients with mild or moderate risk profiles. However, this interpretation should be made cautiously due to the limited sample size, and further studies in larger populations are warranted, as evidenced by the broad 95 % confidence intervals observed for most odds ratios, which reflect the statistical uncertainty associated with small event counts.

Finally, polyurethane-coated implants have emerged as a potential alternative in selected centers, with recent publications reporting favorable outcomes.^25^, ^26^, ^27^, ^28^, ^29^ Nevertheless, robust evidence on their comparative effectiveness is still lacking, and no large-scale data are available to support conclusions regarding their broader adoption or long-term safety. As medicine continues to move toward more personalized approaches, further studies required not only to validate these preliminary results, but also to identify which patient subgroups may benefit most from polyurethane implants compared with biological matrices. Based on our experience, these implants may raise aesthetic concerns in patients with low BMI (particularly <21), especially in the upper breast poles.

## Limitations

This study has several limitations. Although data collection was prospective, the analysis was retrospective. The sample size and follow-up duration were limited, and the single-center design may affect the generalizability of the findings. No multivariable analyses were performed to assess potential associations between risk factors and complications. In addition, the potential confounding effect of surgeon variability and learning curve was not assessed. Although outcomes were not stratified by individual surgeon experience, all procedures were performed in a tertiary, high-volume breast unit by seven board-certified breast surgeons, which may mitigate some of the variability associated with surgeon-dependent factors. Intraoperative assessment of flap viability relied solely on subjective clinical criteria which are highly operator-dependent. The database is currently being expanded to include more cases and longer follow-up, and future studies will address these limitations more comprehensively.

## Conclusions

Prepectoral reconstruction with Exaflex® ADM appears to be a safe and reproducible technique, yielding favorable aesthetic and functional outcomes. However, its routine use may place a financial burden on healthcare systems, particularly in public settings. The authors consider that its use should be prioritized for selected patients (specifically those with risk factors for complications or a borderline pinch test) in whom matrix interposition may help reduce complications, prevent upper pole contour deformities, and promote prosthesis integration. This study represents an initial step toward characterizing the outcomes associated with this matrix. Ongoing follow-up will help confirm these findings. Future research should aim to clearly define the patient subgroups that benefit most from biological matrices, to support evidence-based resource allocation.

## Ethical approval

The study was conducted in accordance with the Declaration of Helsinki. The requirement for informed consent was waived due to the retrospective and anonymized nature of the data.

## Declaration of competing interest

The authors declare no conflicts of interest.
